# Immunostimulant Effect of Egyptian Propolis in Rabbits

**DOI:** 10.1100/2012/901516

**Published:** 2012-05-02

**Authors:** Somya A. Nassar, Amira H. Mohamed, Hamdy Soufy, Soad M. Nasr, K. M. Mahran

**Affiliations:** ^1^Department of Parasitology and Animal Diseases, National Research Center, Dokki, P.O. Box 12622, Giza, Egypt; ^2^Department of Clinical Pathology, Faculty of Veterinary Medicine, Cairo University, P.O. Box 12211, Giza, Egypt

## Abstract

The present experiment was conducted to study the effect of ethanolic extract of Egyptian propolis given alone or in combination with inactivated *Pasteurella multocida* vaccine on rabbits challenged with a virulent strain of *Pasteurella multocida*. Fifty-six New-Zealand rabbits, 6–8 weeks old and non-vaccinated against pasteurellosis, were randomly divided into eight equal groups. The first group was kept as a control for the experiment. The other groups received different treatments with propolis extract, inactivated vaccine, or both. The experiment continued for seven weeks during which clinical signs, body weight, and mortality rate were monitored, and blood samples were collected weekly for evaluating the leukogram, serum biochemistry, and immune response in all groups of animals. At the end of the seventh week, the animals were subjected to challenge with a virulent strain of *Pasteurella multocida*. Two weeks later, tissue specimens were collected from different organs for histopathological examination. Results showed that rabbits of the groups treated with both propolis and the vaccine by different routes appeared healthy after challenge. It has been concluded that alcoholic extract of propolis administrated in combination with inactivated *Pasteurella multocida* vaccine has no adverse effects on the general health conditions and enhances immune response in rabbits.

## 1. Introduction

Rabbits are considered one of the important livestock that provide high quality protein food. One of the important diseases that affect rabbits' production is pasteurellosis. It is a common bacterial disease caused by *Pasteurella multocida* and has been reported as a constant serious and highly contagious disease of domestic rabbits [1]. Pasteurellosis affects rabbits of 4–8 weeks old causing symptoms ranging from fatal septicemia, severe pleuritis, and pneumonia to less severe sequelae such as multiple abscesses, chronic rhinitis, and otitis media. The outcome of any form of the disease is severe economic losses [2].

Control of pasteurellosis in rabbits is accomplished by vaccination against *Pasteurella multocida* infection. Protective immunity can be induced by a live vaccine or an inactivated whole cell vaccine (bactrine). The live vaccine has advantage over the bactrine, though a serious disadvantage is that vaccination with living vaccines, sometimes, results in systemic infection. On the other hand, vaccination of rabbits with bactrines often results in ineffective immunity in the field [3].

Propolis (bee glue) is a resinous hive product, produced by honey bees from various plant sources. It has several biological properties as antimicrobial, antiinflammatory, immunomodulatory, and antioxidant [4–6]. It have been used since ancient times as a medicine because of its biological properties as antiinflammatory, antiallergic properties, anticarcinogenic, antioxidative, antifungal, antiviral, immunostimulant, and for tissue regeneration [7–9]. Administration of propolis alleviated the harmful effects of insecticide, propetamphos [10]. Caffeic acid phenethyl ester (CAPE) is the active component of propolis [11].

The aim of the present study was to evaluate the effect of an extract of Egyptian propolis when administrated either orally or by the subcutaneous (S/C) route with inactivated *Pasteurella multocida* vaccine against experimental challenge with *Pasteurella multocida* strain in rabbits. General performance, leukogram, serum biochemical parameters, and immunological status of rabbits were investigated. Two weeks later from challenge, *postmortem* examination was performed on target organs.

## 2. Materials and Methods

This study was carried out according to guidelines for animal experimentation and approved by the Institutional Animal Care and Use Committee, National Research Centre Animal Care Unit, Dokki, Giza, Egypt.

### 2.1. Animal Used

Fifty-six male New-Zealand rabbits of 1.5-2 kg body weight (B.W.) and 6–8 weeks old were used for the purpose of the present experiment. Rabbits were not previously vaccinated against pasteurellosis, and bacteriological examination of nasopharyngeal swabs proved that they were free from *Pasteurella *infection.

### 2.2. Propolis Extraction

One hundred grams of the resinous material of Egyptian propolis (obtained from Dakahlia Governorate, Egypt) was cut into small pieces and extracted at room temperature with 50 mL of 70% ethanol. Extraction was performed twice with 24 hours interval. The alcoholic extract was evaporated under vacuum at 50°C until dryness. Obtained dried ethanolic extract of propolis (28 g) was suspended in phosphate buffered saline (PBS) (pH 7.2) to obtain 1% stock solution [12]. The dose of propolis used in this experiment was 50 mg/kg B.W. according to Türkez et al. [13].

### 2.3. General Layout of the Experiment

The experiment was carried out at the experimental rabbit unit of Lab Animal House, National Research Center, Dokki, Giza, Egypt. Rabbits were housed in separate cages, fed on a balanced commercial ration and water was available *ad libitum*. The animals were assigned into eight equal groups which were treated with alcoholic extract of propolis alone or in combination with *Pasteurella multocida* inactivated vaccine (obtained from Veterinary Serum and Vaccine Research Institute, Abbasia, Cairo). Propolis was administrated either orally (50 mg/kg B.W./day for one week), or by the subcutaneous (S/C) route (a single dose of 50 mg/kg B.W.). The vaccine was given as a single S/C dose of 2 mL. Treatment of different groups of rabbits was as follows: group (1) injected subcutaneously with 2 mL sterile PBS and was kept as control, group (2) administrated propolis orally, group (3) administrated propolis orally then vaccinated with *Pasteurella multocida* vaccine, group (4) was vaccinated then after one week administrated propolis orally, group (5) was simultaneously vaccinated and administrated propolis orally, group (6) was vaccinated only, group (7) was injected subcutaneously with the vaccine mixed with propolis as an adjuvant, and group (8) was injected subcutaneously with a single dose of propolis. Treatments of propolis and vaccine were repeated after four weeks in all groups. The experiment continued for seven weeks, at the end of which challenge was performed by injection with virulent strain of *Pasteurella multocida*. The strain was obtained from Veterinary Serum and Vaccine Research Institute, Abbasia, Cairo, in the form of lyophilized ampoules. It was activated by culturing in nutrient broth, inoculation in Swiss mice, and reisolation of the organism from heart blood of mice on nutrient agar plates (Difco). *Pasteurella* colonies were suspended in sterile saline and the density was adjusted to contain 5×10^9^ bacterial cell/mL. The suspension was used for S/C inoculation of rabbits in the challenge test [14].

### 2.4. Clinicopathological Investigations

#### 2.4.1. Leukogram and Biochemical Analyses

During the seven-week experimentation time, rabbits were weighed and blood samples were collected weekly. Three blood samples were obtained from the ear vein of each rabbit. The first sample was anticoagulated and used for the determination of the leukogram. A Coulter counter (MEDONIC CA620) was utilized [15]. The second sample was collected for serum separation and determination of serum biochemical constituents and serological studies. Serum biochemical assays included total proteins [16], albumin [17], total cholesterol [18], triglycerides [19], glucose [20], and activities of aminotransferases (AST and ALT) [21], and alkaline phosphatase (ALP) [22]. Serum globulins were determined by subtracting the value of serum albumin from the value of serum total proteins. Commercial diagnostic kits from Biomerieux, France, and Quimica Clinica Aplicada (QCA), Amposta, Spain, were used for assay of serum biochemical parameters.

### 2.5. Immunological Studies

#### 2.5.1. Humeral Immune Response

Serum samples were utilized also for estimation of the humeral immune response against *Pasteurella multocida* antigen in rabbits. The enzyme-linked immunosorbant assay (ELISA) was used according to Shu et al. [23]. A positive result was considered when the absorbance value was equal to or more than the cut-off value. The cut-off value equals double fold the mean value of negative sera.

#### 2.5.2. Cellular Immune Response (Lymphocyte Proliferation Assay (BrdU))

The third blood sample was collected in sterile heparinized tubes from groups 1, 6, 7, and 8 at the 1st, 2nd, and 4th weeks after vaccination. Blood was used for separation of mononuclear leukocytes for the lymphocyte proliferation assay (BrdU) to measure the cellular immune response of rabbits against *Pasteurella multocida*. A test kit from Roche Diagnostics, Germany, was used.

### 2.6. Challenge Assay

At the end of the experiment (7th week), experimental rabbits were challenged by S/C injection of 0.2 mL/rabbit of broth culture of virulent *Pasteurella multocida*. Reisolation and identification of *Pasteurella* organisms were done from the heart blood of rabbits died after challenge [14].

### 2.7. *Postmortem* Examination

Two weeks later from challenge test, *Postmortem* investigation was performed on target organs (heart, trachea, lungs, liver, kidneys, and spleen).

### 2.8. Statistical Analysis

Statistical analysis was performed on the collected data for the mean and standard error of the mean. Significance of the results was determined using two-way analysis of variance followed by Duncan's multiple range tests. Differences were considered significant at *P* < 0.05 level [24] using SPSS version 10 computer programme.

## 3. Results

### 3.1. Clinical Signs

Rabbits of different groups appeared normal before challenge with the virulent strain of *Pasteurella multocida*. One day after challenge, rabbits of the control group (group 1) showed acute signs of the disease in the form of depression, sneezing, and respiratory manifestations. Some rabbits showed nervous manifestations and sudden death. Mortality rate in this group reached 100%. Rabbits treated with either propolis or the vaccine only (groups 2, 6, and 8) showed less severe clinical signs than the control group. Some rabbits of group (6) which received the vaccine only showed superficial multiple abscesses (the chronic form of the disease). Mortality rate was 57.14% in groups 2 and 8 and 28.57% in group 6. Rabbits treated with both propolis and the vaccine by different regimens (groups 3, 4, 5, and 7) were apparently healthy and showed no mortalities after challenge. Body weight of different experimental groups did not change significantly compared to control group.

### 3.2. Leukogram

**Figure 1:**
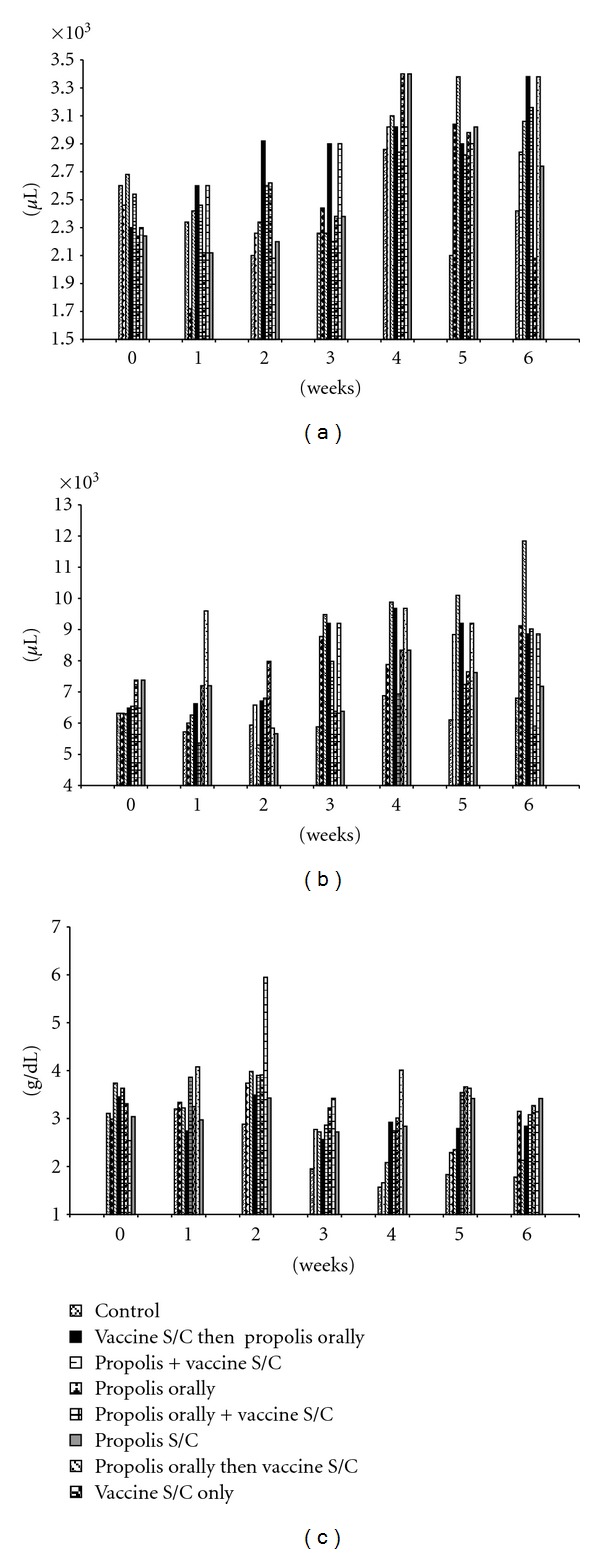
Heterophil (a), lymphocyte (b) counts, and serum globulins (c) in different experimental groups of rabbits received propolis and vaccine treatments for six weeks.

Total leukocytes' count was elevated in all experimental groups except group 8 (received S/C propolis only) compared to control group. Elevation of total leukocytes was associated with elevation of lymphocytes and heterophils (Table 1, Figures 1(a) and 1(b)). Monocytes and eosinophils revealed variable changes in different experimental groups.

### 3.3. Biochemical Changes

Values of serum total protein were elevated in groups 7 and 8 (received S/C propolis only or with the vaccine) throughout the experimentation time. Values were elevated in the other groups at different times between the 3rd and 6th week of the experiment. Serum globulin values were elevated in all vaccinated groups (4, 5, 6, and 7) and in the group treated with propolis subcutaneously (8th group) from the 2nd to the 6th week of the experiment (Table 2, Figure 1(c)).

**Figure 2:**
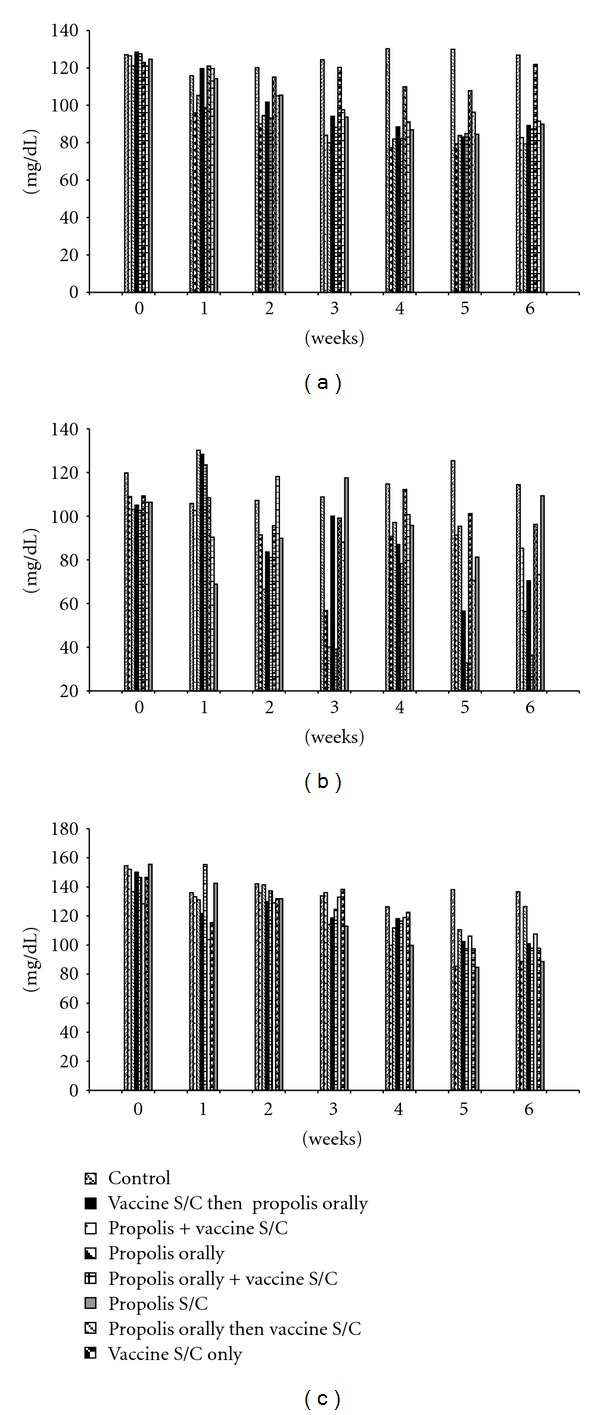
Serum glucose (a), total cholesterol (b), and triglycerides (c) in different experimental groups of rabbits received propolis and vaccine treatments for six weeks.

Serum glucose values were decreased starting from the 1st or 2nd week to the end of the experiment in all groups received propolis in their treatment. The decrease was from the 4th week to the end of the experiment in the group received the vaccine only (group 6) (Figure 2(a)).

Cholesterol values were decreased from the 2nd to the 6th week of the experiment in groups 2, 3, 4, and 5, and at different times of the experiment in groups 6, 7 and 8. Values of triglycerides were decreased from the 4th to the 6th week in all experimental groups (Figures 2(b) and 2(c)).

Activity of serum enzymes was decreased in different experimental groups. AST and ALT activity was elevated in groups 6 and 7, respectively, between the 2nd and 4th week of the experiment. In the other groups AST, and ALT activity was elevated occasionally. Changes in ALP activity were less marked in different experimental groups.

### 3.4. Immunological Results

Examination of the humeral immune response of rabbits by the enzyme-linked immunosorbant assay (ELISA) revealed elevation of serum antibodies in all groups, but group 2 and 8 received propolis by the oral or the S/C route, from the 2nd to the 7th week of the experiment (Table 3).

Cellular immune response measured by the lymphocyte proliferation assay in groups 6, 7, and 8 at the end of the 1st, 2nd, and 4th weeks of the experiment revealed positive reaction from the 1st to the 4th week in group 6 (vaccinated group), at the 2nd and 4th weeks in group 7, and at the 4th week in group 8. The response in groups 7 and 8 was less than that in group 6 (Table 4).

### 3.5. *Postmortem* Investigations

*Postmortem* findings of control rabbits group challenged with *Pasteurella multocida *strain showed lesions of acute pasteurellosis in the form of severe rhinitis with nasal discharges, congested blood vessels with S/C hemorrhage, presence of blood in the thorax and abdomen, severe congestion of trachea, lungs and heart, necrotic foci in the liver, and congested friable kidneys. Rabbits of groups 2 and 8 administrated propolis only showed S/C hyperemic patches, congested heart, trachea and lungs, enlarged liver with necrotic foci, congested and enlarged spleen. Rabbits administrated the vaccine only (group 6) showed less severe lesions of the disease represented by presence of multiple lung abscesses, congestion of the lungs, enlarged urinary bladder which was filled with urine and salts. Rabbits of the groups administrated propolis and vaccine appeared normal when scarified 15 days after challenge. Some rabbits showed multiple S/C abscesses in the front leg, and in the neck.

## 4. Discussion

A large body of investigators has reported on the use of immune stimulants for enhancement of the immune response during vaccination. Propolis is a resinous hive product collected by honey bees from various plant sources. It contains more than 160 constituents that have several biological and pharmacological properties such as antimicrobial, antiinflammatory, immunomodularity, and antioxidant effects [25–28].

The present work was carried out to evaluate the effect of an ethanolic extract of Egyptian propolis as immunostimulant to an inactivated formalized *Pasteurella multocida* vaccine used to immunize rabbits. Propolis was administrated either orally or by S/C injection with, or without the vaccine. Evaluation was assessed by observation of the clinical signs, clinicopathological and immunological investigations. At the end of treatment, groups of rabbits were challenged with a virulent strain of *Pasteurella multocida *and evaluated by observation of the clinical signs, mortality rate, and *postmortem *investigation.

Experimental rabbits appeared healthy during the time of the experiment before challenge. Investigation of the leukogram revealed leukocytosis in almost all experimental groups except those treated with the vaccine (group 6) or propolis only (group 8). Leukocytosis was associated with heterophilia and lymphocytosis. The obtained results may indicate an immune-stimulatory effect of propolis when combined with the vaccine [29, 30]. It has been reported that propolis has a direct regulatory effect on the basic functional properties of immune cells [31]. Artepillin C which is one of propolis components has been described to activate the immune system by increasing phagocytic activity as well as number of lymphocytes [32]. Propolis extract may increase production of the lymphocyte activating factor IL-1 which enhances B- and T-cell proliferation [33, 34].

Total protein values were elevated especially in groups treated with the vaccine and propolis S/C, or propolis S/C (groups 7 and 8, resp.). The elevation was associated with increase of globulin values. Results pointed out to a nonspecific immunostimulant effect of propolis alone or as adjuvant to the vaccine [34, 35] and a specific immune response induced by *Pasteurella multocida *vaccine [36, 37].

Results of serum lipids revealed that the ethanolic extract of propolis has a decreasing effect on serum total cholesterol and triglycerides which may be attributed to the presence of flavonoids, steroids, phenolic acids, and their esters among propolis constituents [12, 38]. These compounds may affect directly lipid metabolism leading to decrease of cholesterol and triglycerides in blood [39]. Such results are confirmed by the findings of Badawi [40] and Ali [41]. Fuliang et al. [42] reported that oral administration of propolis significantly lowered total cholesterol and triglycerides in serum of rats. Alves et al. [43] reported that the hypocholesterolemic effect of propolis could be a result of a direct effect on the liver or an indirect effect through thyroid hormones which affect reactions in almost all the pathways of lipid metabolism.

Along the period of experiment, the activity of serum enzyme AST was decreased in different experimental groups except in group 6 (vaccinated group), where the elevation was recorded during the 2nd to the 4th week of the experiment. The present results agree with Hegazi et al. [12], and Talas and Gulhan [38]. Badawi [40] found that administration of propolis to rats in a dose of 150–1500 mg/kg B.W. caused slight inhibition of the activity of transferase enzymes. Ali [41] reported no apparent change in serum AST activity due to single dose of propolis (100 mg) in rats. The result of the present study indicated that administration of propolis had no toxic effect on rabbits. Activity of serum ALT followed a similar pattern to that of AST. Similar results were observed by Kleinrok et al. [44] and Eraslan et al. [39]. Oliveira et al. [45], on the other hand, reported that crude propolis extract did not cause significant alterations in the serum enzymes alanine and aspartate aminotransferase activities.

Results of serum ALP activity revealed significant decrease in groups 2 through 6 during the period from the 1st to the 3rd week of the experiment which may be due to the action of propolis as reducing agent to ALP. The present results agree with Hegazi et al. [12].

Values of serum glucose were decreased in all groups administrated propolis. It has been described that this decrease was related to inhibition of the activity of intestinal maltase by propolis [39].

The ELISA positive titer of *Pasteurella multocida* antibodies in group 6 (vaccinated only) agrees with Borkowska et al. [36, 37]. Antibody titers in groups treated with the vaccine and propolis by different routes were higher than that of the vaccinated group and may be attributed to the ability of propolis for modulating the synthesis of antibodies [46]. Previous reports stated that the ethanolic extract of propolis increased antibody production [33] and has potent effect on different cells of innate immune response [47]. CAPI which is one of propolis components increase T- lymphocyte proliferation as well as secretion of IL-1 and IL-2 by splenocytes [48]. Chu [34] mentioned that propolis could activate antigen presenting cells (e.g., macrophages) to produce cytokines which activate T and B lymphocytes. Ansorge et al. [31] and Cuesta et al. [49] were of the opinion that propolis stimulated nonspecific and specific immunity factors.

Positive titers of lymphocyte proliferation were only recorded at the 2nd and 4th weeks in groups 6 and 7. This result may be attributed to that inactivated vaccine enhances mainly humeral immune response and specially IgG [50, 51]. The weak effect of propolis on lymphocyte proliferation may be related to the inhibitory effect of CAPI on some transcription factors [52]. Similar findings were previously reported by Hu et al. [53] and Paulino et al. [54].

In conclusion, the ethanolic extract of Egyptian propolis, when administrated in combination with formalized inactivated *Pasteurella multocida *vaccine in rabbits' enhanced specific and nonspecific immune response, revealed no toxic effect and reduced the severity of adverse clinical signs, and mortality rate. The present experimental trial can encourage the use of propolis as an immunostimulant with human and animal vaccines.

## Figures and Tables

**Table 1:** Total leukocytes count (×10^3^/*μ*L) in different experimental groups of rabbits received propolis and vaccine treatments for six weeks (mean ± SE).

Groups Period (week)	Control (1)	Propolis orally (2)	Propolis orally then vaccine S/C (3)	Vaccine S/C then propolis orally (4)	Propolis orally + vaccine S/C (5)	Vaccine S/C only (6)	Propolis + vaccine S/C (7)	Propolis S/C (8)
1	8.85^bd^ ±0.55	7.98^d^ ±0.40	8.93^bd^ ±0.52	9.60^bc^ ±0.51	8.24^cd^ ±0.13	9.68^b^ ±0.45	12.50^a^ ±0.31	9.82^b^ ±0.45
2	8.42^bc^ ±0.57	9.10^bc^ ±0.76	7.82^c^ ±0.35	8.31^bc^ ±0.37	9.80^ab^ ±0.90	11.06^a^ ±0.24	8.22^bc^ ±0.28	8.14^bc^ ±0.33
3	8.89^b^ ±0.61	11.60^a^ ±0.56	12.43^a^ ±0.78	12.95^a^ ±0.37	11.50^a^ ±0.48	8.96^b^ ±0.23	12.78^a^ ±0.25	9.28^b^ ±0.30
4	10.32^c^ ±0.27	11.12^bc^ ±1.28	13.66^a^ ±0.76	13.18^ab^ ±0.41	10.35^c^ ±0.39	12.28^a-c^ ±0.56	13.37^a^ ±0.35	12.44^a-c^ ±0.78
5	8.57^e^ ±0.24	12.56^b^ ±0.25	14.18^a^ ±0.62	12.12^bc^ ±0.45	10.44^d^ ±0.42	11.18^cd^ ±0.32	12.78^b^ ±0.40	11.36^cd^ ±0.27
6	9.90^c^ ±1.22	12.36^b^ ±0.33	16.54^a^ ±0.41	12.84^b^ ±0.49	12.40^b^ ±0.73	8.65^c^ ±0.23	12.86^b^ ±0.82	10.34^c^ ±0.39

Means followed by different superscripts (a, b, c, d, e) within the same row are significantly different at *P* < 0.05.

SE: standard error.

**Table 2:** Serum total proteins (g/dL) in different experimental groups of rabbits received propolis and vaccine treatments for six weeks (mean ± SE).

Groups Period (week)	Control (1)	Propolis orally (2)	Propolis orally then vaccine S/C (3)	Vaccine S/C then propolis orally (4)	Propolis orally + vaccine S/C (5)	Vaccine S/C only (6)	Propolis + vaccine S/C (7)	Propolis S/C (8)
1	7.23^b-c^ ±0.30	6.95^bc^ ±0.91	6.23^c^ ±0.39	6.91^bc^ ±0.64	7.02^bc^ ±0.46	7.52^b^ ±0.30	8.51^a^ ±0.39	8.77^a^ ±0.20
2	6.32^c^ ±0.44	7.04^bc^ ±0.70	7.43^bc^ ±0.34	7.40^bc^ ±0.19	7.35^bc^ ±0.14	6.97^c^ ±0.38	9.45^a^ ±0.65	7.98^b^ ±0.12
3	5.34^d^ ±0.28	5.19^cd^ ±0.91	7.32^a-c^ ±0.60	7.16^bc^ ±0.22	6.57^cd^ ±0.30	8.11^a^ ±0.20	8.88^a^ ±0.46	7.79^ab^ ±0.17
4	4.99^d^ ±0.31	7.11^bc^ ±0.19	6.15^c^ ±0.49	6.72^c^ ±0.23	7.66^b^ ±0.16	6.32^c^ ±0.10	9.20^a^ ±0.53	8.19^b^ ±0.41
5	4.95^d^ ±0.25	7.96^bc^ ±0.26	7.13^bc^ ±0.32	7.02^bc^ ±0.23	8.93^a^ ±0.28	8.64^ab^ ±0.38	9.35^a^ ±0.20	8.40^b^ ±0.03
6	5.15^c^ ±0.31	8.21^a^ ±0.03	5.23^c^ ±0.10	6.27^bc^ ±0.12	6.21^bc^ ±0.30	8.59^a^ ±0.53	8.52^a^ ±0.35	7.49^b^ ±0.17

Means followed by different superscripts (a, b, c, d, e) within the same row are significantly different at *P* < 0.05.

SE: standard error.

**Table 3:** Enzyme-linked immunosorbant assay (ELISA) in different experimental groups of rabbits received propolis and vaccine treatments from the second till the seven week (mean ± SE).

Groups Period (week)	Control (1)	Propolis orally (2)	Propolis orally then vaccine S/C (3)	Vaccine S/C then propolis orally (4)	Propolis orally + vaccine S/C (5)	Vaccine S/C only (6)	Propolis + vaccine S/C (7)	Propolis S/C (8)
2	0.136 ±0.01	0.124 ±0.04	0.504 ±0.02	0.502 ±0.05	0.518 ±0.03	0.531 ±0.06	0.522 ±0.05	0.144 ±0.01
3	0.131 ±0.00	0.118 ±0.03	0.412 ±0.03	0.532 ±0.00	0.464 ±0.01	0.493 ±0.01	0.441 ±0.04	0.126 ±0.04
4	0.149 ±0.00	0.201 ±0.02	0.350 ±0.02	0.371 ±0.01	0.494 ±0.07	0.285 ±0.01	0.421 ±0.03	0.152 ±0.01
5	0.154 ±0.01	0.144 ±0.01	0.344 ±0.01	0.516 ±0.03	0.483 ±0.00	0.353 ±0.03	0.455 ±0.01	0.154 ±0.03
6	0.151 ±0.00	0.127 ±0.00	0.361 ±0.02	0.409 ±0.02	0.561 ±0.01	0.402 ±0.03	0.533 ±0.04	0.149 ±0.02
7	0.135 ±0.01	0.150 ±0.00	0.403 ±0.02	0.463 ±0.02	0.408 ±0.01	0.471 ±0.01	0.414 ±0.02	0.138 ±0.01

*Cut-off value*	0.290							

**Table 4:** Lymphocyte proliferation assay (BrdU) in different experimental groups of rabbits received propolis and vaccine treatments for six weeks (mean ± SE).

Groups Period (week)	Control (1)	Vaccine S/C only (6)	Propolis + vaccine S/C (7)	Propolis S/C (8)
1	0.09^b^ ±0.01	0.19^a^ ±0.03	0.14^ab^ ±0.02	0.10^b^ ±0.02
2	0.10^b^ ±0.02	0.21^a^ ±0.06	0.23^a^ ±0.03	0.10^b^ ±0.03
4	0.06^c^ ±0.03	0.26^a^ ±0.04	0.26^a^ ±0.02	0.14^b^ ±0.03

Means followed by different superscripts (a, b, c, d, e) within the same row are significantly different at *P* < 0.05.

SE: standard error.
